# New Postmortem Perspective on Emerging SARS-CoV-2 Variants of Concern, Germany

**DOI:** 10.3201/eid2903.221297

**Published:** 2023-03

**Authors:** Fabian Heinrich, Tobias Huter, Sophie Mertens, Philine Lange, Jessica Vering, Axel Heinemann, Dominik Sebastian Nörz, Armin Hoffmann, Martin Aepfelbacher, Benjamin Ondruschka, Susanne Krasemann, Marc Lütgehetmann

**Affiliations:** University Medical Center Hamburg-Eppendorf, Hamburg, Germany

**Keywords:** COVID-19, respiratory infections, severe acute respiratory syndrome coronavirus 2, SARS-CoV-2, SARS, coronavirus disease, zoonoses, viruses, coronavirus, VOC, variants of concern, B.1.1.7, Alpha variant, B.1.617.2, Delta variant, B.1.1.529, Omicron variant, respiratory infections, organ tropism, viral load, pulmonary damage, Germany

## Abstract

We performed autopsies on persons in Germany who died from COVID-19 and observed higher nasopharyngeal SARS-CoV-2 viral loads for variants of concern (VOC) compared with non-VOC lineages. Pulmonary inflammation and damage appeared higher in non-VOC than VOC lineages until adjusted for vaccination status, suggesting COVID-19 vaccination may mitigate pulmonary damage.

SARS-CoV-2 emerged in 2020, and after rapid global spread, virus variants emerged that had adaptation or immune evasion mutations and were classified as variants of concern (VOCs). Although the first VOCs, Alpha (B.1.1.7) and Delta (B.1.617.2), showed enhanced transmissibility (*1*), Omicron (B.1.1.529) lineages carry mutations that provide strong immune evasion after infection with previous lineages or mRNA vaccination (*2*). Because autopsy data are lacking, differences in SARS-CoV-2–related pulmonary disease and tropisms have not been well studied. In this study, we performed full autopsies of persons who died from COVID-19 and conducted comprehensive analyses to characterize COVID-19–related cases macroscopically and microscopically.

All corpses admitted to the Institute of Legal Medicine (n = 8,578) and crematoria (n = 1,705) in Hamburg, Germany, during March 3, 2020–March 31, 2022, were screened for SARS-CoV-2 mRNA by quantitative reverse transcription PCR (*3*). We found a total of 808 SARS-CoV-2 RNA-positive corpses; median monthly prevalence was 6.54% in 2020, 5.28% in 2021, and 12.50% in 2022 (Figure, panel A). In comparison, the median monthly prevalence of SARS-CoV-2 in Hamburg’s general population was 0.10% in 2020, 0.37% in 2021, and ≈5.20% in 2022 (https://de.statista.com/statistik/daten/studie/1106006/umfrage/entwicklung-der-fallzahl-des-coronavirus-in-hamburg). A considerably higher prevalence of virus in deceased persons than in the general population can be explained by an older age in postmortem cohorts, targeted transport of clinically suspected or confirmed COVID-19 deaths to the Institute of Legal Medicine in 2020, and underreporting of overall SARS-CoV-2 prevalence because of limited availability of molecular testing.

**Figure:**
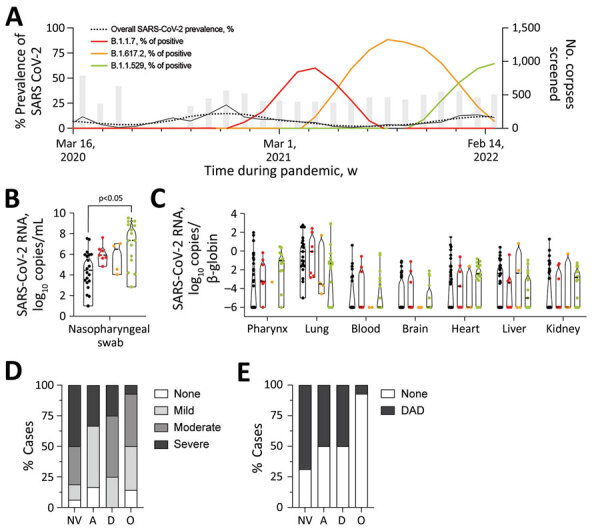
Prevalence of SARS-CoV-2 variants, pulmonary inflammation, and diffuse alveolar damage in corpses autopsied during 2020 and 2022 at the Institute of Legal Medicine and crematoria in Hamburg, Germany. A) Overall prevalence of corpses positive for SARS-CoV-2 mRNA and prevalence of B.1.1.7 (Alpha), B.1.617.2 (Delta), and B.1.1.529 (Omicron) variants of concern as the percentage of SARS-CoV-2 mRNA-positive corpses are depicted; 3-point centered moving averages are shown. Gray bars indicate monthly number of corpses screened for SARS-CoV-2 mRNA. B, C) Number of SARS-CoV-2 mRNA copies in different autopsy specimens. Median and interquartile ranges of viral mRNA loads were stratified according to virus variants in nasopharyngeal swabs (B) and different organs (C). Nasopharyngeal and organ viral loads for non-VOC and B.1.1.7 were published in part previously (*7*,*8*). Black dots are non-VOC lineages. Pairwise comparisons were performed by using the Kruskal-Wallis H test and Dunn post-hoc analysis. D, E) Percentage of cases that had pulmonary inflammation or alveolar damage caused by different SARS-CoV-2 lineages: NV, Non-VOC lineage; A, Alpha B.1.1.7 lineage; D, Delta B.1.617.2 lineage; O, Omicron B.1.1.529 lineage. D) We scored microscopic pulmonary inflammation as follows: none, 0; mild, 1; moderate, 2; or severe, 3 on the basis of a Likert-like scale and calculated percentages of cases within each group for each SARS-CoV-2 variant. E) Percentage of corpses infected with non-VOC lineages (n = 16) and the VOC lineages B.1.1.7 (Alpha, n = 6), B.1.617.2 (Delta, n = 4), and B.1.1.529 (Omicron, n = 14) that had DAD in lungs. DAD, diffuse alveolar damage; VOC, variant of concern.

We further characterized SARS-CoV-2 RNA-positive samples by using multiplexed typing quantitative reverse transcription PCR (*4*,*5*). The occurrence of VOCs among corpses (B.1.1.7 for December 2020–March 2022, B.1.617.2 beginning in May 2021, and B.1.1.529 beginning in November 2021) reflected their occurrence within the general population but had a 2–4 week delay (https://www.leibniz-liv.de/de/aktuelles/covid-19/daten-der-hamburg-surveillance-plattform).

Of the 808 COVID-19–associated deaths (determined by postmortem SARS-CoV-2 RNA detection), we autopsied 49 corpses and collected multiorgan tissue samples for more detailed analyses. We included 23/49 consecutive deceased persons infected with non-VOC lineages and 26/49 consecutive deceased persons infected with VOCs (B.1.1.7, n = 7; B.1.617.2, n = 4; and B.1.1.529, n = 15) (Table). We processed formalin-fixed paraffin-embedded tissue for histologic and immunohistochemical staining and cryopreserved tissue for molecular analysis as previously described (*3*,*6*).

**Table:** Baseline characteristics of persons whose deaths were associated with SARS-CoV-2 infection, grouped according to SARS-CoV-2 virus variant in study of new postmortem perspective on emerging SARS-CoV-2 variants of concern, Germany*

Variable	Non-VOC lineages	B.1.1.7†	B.1.617.2†	B.1.1.529†	p value	Cohort total
No. corpses	23	7	4	15	NA	49
Age, y, median (IQR)	76.0 (70.0–85.0)	75.0 (52.0–77.0)	50.5 (42.5–70.0)	75.0 (58.0–87.0)	0.29	75.0 (63.0–85.0)
Sex					0.31	
M	15 (65.2)	4 (57.1)	2 (50.0)	5 (33.3)	NA	26 (53.1)
F	8 (34.8)	3 (42.9)	2 (50.0)	10 (66.7)	NA	23 (46.9)
BMI, kg/m^2^, median (IQR)	25.3 (20.7–31.9)	29.5 (26.1–34.8)	38.4 (16.5–42.9)	22.6 (18.8–23.6)	0.02	24.8 (20.7–31.0)
COVID-19 deaths	20 (87.0)	6 (85.7)	3 (75.0)	3 (20.0)	<0.0001	32 (65.3)
No. underlying conditions, median (IQR)	4.0 (3.0–4.0)	2.0 (2.0–3.0)	3.0 (2.0–5.0)	4.0 (2.0–5.0)	0.24	3.5 (2.0–4.0)
Place of death					0.23	
Home	5 (21.7)	3 (42.9)	0 (0.0)	5 (33.3)	NA	13 (26.5)
Normal ward	9 (39.1)	2 (28.6)	2 (50.0)	2 (13.3)	NA	15 (30.6)
ICU	5 (21.7)	2 (28.6)	2 (50.0)	2 (13.3)	NA	11 (22.4)
Other	4 (17.4)	0 (0.0)	0 (0.0)	6 (40.0)	NA	10 (20.4)
PMI, d, median (IQR)	1.0 (0.0–1.0)	3.0 (1.0–6.0)	1.0 (1.0–1.5)	0.0 (0.0–1.0)	0.03	1.0 (0.0–2.0)
Vaccination	0 (0.0)	1 (7.7)	1 (7.7)	11 (84.6)	<0.0001	13 (27.1)

*Corpses were admitted to the Institute of Legal Medicine, University Medical Center Hamburg-Eppendorf, Hamburg, Germany, for autopsy during 2020–22. Values are no. (%) unless otherwise noted. BMI, body mass index; ICU, intensive care unit; IQR, interquartile range; NA, not applicable; PMI, postmortem interval (time elapsed from death until cooling at 4°C); VOC, variant of concern. †VOC lineages B.1.1.7 (Alpha), B.1.617.2 (Delta), and B.1.1.529 (Omicron).

The median nasopharyngeal SARS-CoV-2 RNA load was 5.82 (interquartile range [IQR] 4.08–7.31) log_10_ copies/mL (Figure, panel B). Nasopharyngeal and organ viral loads for non-VOC and B.1.1.7 were published in part previously (*7*,*8*). We observed strong evidence for a difference in mean nasopharyngeal viral loads by virus variant (p = 0.01); by using multiple comparisons, we observed a difference in means between B.1.1.529 and non-VOC lineages (p = 0.002; Figure, panel B). This result supports increased infectivity of B.1.1.529 compared with non-VOC lineages (*9*). An association between nasopharyngeal virus load and virus variant at the 5% significance level did not persist in a multivariable model adjusted for the deceased’s vaccination status, which might be because of the small sample size (Appendix Tables 1, 2). Of note, the pulmonary virus load was strongly associated with viremia (odds ratio 2.21, 95% CI 1.34–3.63; p = 0.002) and mRNA detection in peripheral organs (odds ratio 1.54, 95% CI 1.10–2.16; p = 0.01) in univariable logistic regression models. However, normalized SARS-CoV-2 RNA loads in peripheral organs did not differ between virus variants (Figure, panel C).

Experienced pathologists performed single-blind histologic assessments. We detected SARS-CoV-2 nucleocapsid protein in the lungs of 25/41 (61%) cases. Using a Likert-like scale, we found the median abundance of SARS-CoV-2 nucleocapsid protein (0, not detected; 1, low abundance; 2, intermediate abundance; 3, high abundance) was 1 (IQR 0–2) for non-VOC lineage, 1.5 (IQR 1–2) for B.1.1.7, 0.5 (IQR 0–1) for B.1.617.2, and 0 (IQR 0–1) for B.1.1.529 cases (p = 0.03) (Appendix Figure).

We detected mild to strong inflammatory changes in the lungs of 36/40 (90%) cases and microscopic signs of diffuse alveolar damage (DAD), indicating acute respiratory distress syndrome, in 17/40 (43%) cases (Figure, panels D, E). As in recent animal experiments (*10*), pulmonary changes, such as inflammation and DAD, were associated with virus variant at the 5% level (Appendix Tables 3, 4) but not with nasopharyngeal or pulmonary viral load (inflammatory changes, p>0.05; DAD, p>0.05). An association between virus variants and inflammatory changes or DAD at the 5% level did not persist in a multivariable model adjusted for the deceased’s vaccination status (Appendix Tables 5–7).

In conclusion, our data confirm higher SARS-CoV-2 mRNA loads in nasopharynges of deceased persons who were infected with the B.1.1.529 VOC lineage, but we observed no differences in pulmonary or tertiary organ viral loads. However, pulmonary inflammation appeared higher and DAD more frequent in non-VOC than VOC lineages until adjustment for vaccination status. Our results suggest that prior vaccination, rather than reduced virulence of virus variants, might convey protection against pulmonary inflammation and acute respiratory distress syndrome during SARS-CoV-2 infections.

Appendix Additional information for new postmortem perspective on emerging SARS-CoV-2 variants of concern, Germany.
