# Supplementary material for: New Postmortem Perspective on Emerging SARS-CoV-2 Variants of Concern, Germany
Source: Emerg Infect Dis. 2023 Mar;29(3):652–6. doi: 10.3201/eid2903.221297 (PMC9973696; doi:10.3201/eid2903.221297)
Supplement: Appendix — Additional information for new postmortem perspective on emerging SARS-CoV-2 variants of concern, Germany. [file 22-1297-Techapp-s1.pdf]

Article DOI: https://doi.org/10.3201/eid2903.221297

EID cannot ensure accessibility for supplementary materials supplied by authors. Readers who have difficulty accessing supplementary content should contact the authors for assistance.

New Postmortem Perspective on Emerging SARS-CoV-2 Variants of Concern, Germany

Appendix

Appendix Table 1. Multivariable analysis of nasopharyngeal viral loads and SARS-CoV-2 variants in study of new postmortem perspective on emerging SARS-CoV-2 variants of concern, Germany*

Virus variant (reference: non-VOC lineage)	β coefficient (95% CI)†	p value
B.1.1.7	1.94 (0.47–3.41)	0.01
B.1.617.2	1.47 (–0.46–3.40)	0.13
B.1.1.529	2.93 (1.19–4.66)	0.001

*Linear regression model where nasopharyngeal viral load (log copies/mL) was the dependent variable and SAR-CoV-2 variant was the independent variable. Variants were determined by multiplexed typing quantitative reverse transcription PCR. VOC, variant of concern.
†95% CIs are robust for heteroscedasticity. Global F-test, p = 0.01.

Appendix Table 2. Multivariable analysis of nasopharyngeal viral loads, SARS-CoV-2 variants, and vaccination status in study of new postmortem perspective on emerging SARS-CoV-2 variants of concern, Germany*

Virus variant (reference: non-VOC lineage)	β coefficient (95% CI)†	p value
B.1.1.7	1.43 (–0.04–2.91)	0.06
B.1.617.2	1.10 (–0.76–2.95)	0.24
B.1.1.529	1.83 (–0.63–4.28)	0.14
Vaccination (reference: none)	1.50 (–0.43–3.43)	0.13

*Linear regression model where nasopharyngeal viral load (log copies/mL) was the dependent variable and SAR-CoV-2 variant and vaccination status (0/1) were independent variables. Variants were determined by multiplexed typing quantitative reverse transcription PCR. VOC, variant of concern.
†95% CIs are robust for heteroscedasticity.

Appendix Table 3. Multivariable analysis of inflammatory changes and SARS-CoV-2 variants in study of new postmortem perspective on emerging SARS-CoV-2 variants of concern, Germany*

Virus variant (reference: non-VOC lineage)	β coefficient (95% CI)†	p value
B.1.1.7	−0.75 (–1.84–0.34)	0.17
B.1.617.2	−0.25 (–1.15–0.65)	0.58
B.1.1.529	−0.82 (–1.49 to –0.15)	0.02

*Linear regression model where inflammatory changes were the dependent variable and SAR-CoV-2 variants were the independent variables. Variants were determined by multiplexed typing quantitative reverse transcription PCR. VOC, variant of concern.
†95% confidence intervals are robust for heteroscedasticity. Global F-test, p = 0.10.

Appendix Table 4. Multivariable logistic regression analysis of diffuse alveolar damage and SARS-CoV-2 variants in study of new postmortem perspective on emerging SARS-CoV-2 variants of concern, Germany*

Virus variant (reference: non-VOC lineage)	β coefficient (95% CI)†	p value
B.1.1.7	–0.79 (–2.73–1.15)	0.43
B.1.617.2	–0.79 (–3.04–1.47)	0.49
B.1.1.529	–3.35 (–5.67–1.03)	0.01

*Diffuse alveolar damage was the dependent variable and SAR-CoV-2 variants were the independent variables. Variants were determined by multiplexed typing quantitative reverse transcription PCR. Composite Wald test, p = 0.05. VOC, variant of concern.
†95% CIs were calculated on the basis of robust standard errors.

Appendix Table 5. Multivariable analysis of inflammatory changes, SARS-CoV-2 variants, and vaccination status in study of new postmortem perspective on emerging SARS-CoV-2 variants of concern, Germany*

Virus variant (reference: non-VOC lineage)	β coefficient (95% CI)†	p value
B.1.1.7	–0.65 (–1.92–0.62)	0.30
B.1.617.2	–0.02 (–0.75–0.71)	0.95
B.1.1.529	–0.17 (–1.01–0.67)	0.68
Vaccination (reference: none)	–0.91 (–1.67 to –0.15)	0.02

*Linear regression model where inflammatory changes were the dependent variable and SAR-CoV-2 variants and vaccination status were independent variables. Variants were determined by multiplexed typing quantitative reverse transcription PCR. VOC, variant of concern.
†95% CIs are robust for heteroscedasticity.

Appendix Table 6. Multivariable logistic regression analysis of diffuse alveolar damage, SARS-CoV-2 variants, and vaccination status in study of new postmortem perspective on emerging SARS-CoV-2 variants of concern, Germany*

Virus variant (reference: non-VOC lineage)	β coefficient (95% CI)†	p value
B.1.1.7	−1.19 (−3.31–0.92)	0.27
B.1.617.2	−0.10 (−2.77–2.58)	0.94
B.1.1.529	−1.89 (−4.43–0.66)	0.15
Vaccination (reference: none)‡	ND	NA

*Diffuse alveolar damage was the dependent variable and SAR-CoV-2 variants and vaccination status were the independent variables. Variants were determined by multiplexed typing quantitative reverse transcription PCR. NA, not applicable; ND, not done; VOC, variant of concern.
†95% CIs were calculated on the basis of robust standard errors.
‡Vaccination status predicted the outcome perfectly because of data separation and small sample size.

Appendix Table 7. Baseline characteristics of persons whose deaths were associated with SARS-CoV-2 infection, grouped according to vaccination status in study of new postmortem perspective on emerging SARS-CoV-2 variants of concern, Germany*

Characteristics	Unvaccinated	Vaccinated	p value	Overall cohort
No. patients†	35	13	NA	48
Age, y, median (IQR)	75.0 (55.0–82.0)	85.0 (65.0–87.0)	0.03	75.0 (60.5–85.0)
Sex			0.18	NA
M	21 (60.0)	5 (38.5)	NA	26 (54.2)
F	14 (40.0)	8 (61.5)	NA	22 (45.8)
BMI, kg/m^2, median (IQR)	25.5 (20.7–34.6)	23.0 (19.3–24.8)	0.08	24.2 (20.5–31.4)
COVID-19 cause of death	27 (77.1)	4 (30.8)	0.01	31 (64.6)
No. pre-existing medical conditions, median (IQR)	3.5 (2.0–4.0)	4.0 (3.0–5.0)	0.24	4.0 (2.0–4.0)
Place of death			0.07	NA
Home	10 (28.6)	2 (15.4)	NA	12 (25.0)
Normal ward	11 (31.4)	4 (30.8)	NA	15 (31.2)
ICU	10 (28.6)	1 (7.7)	NA	11 (22.9)
Other	4 (11.4)	6 (46.2)	NA	10 (20.8)
PMI, d, median (IQR)	1.0 (0.0–2.0)	0.0 (0.0–1.0)	0.13	1.0 (0.0–2.0)
Pulmonary inflammation (scores 0–3), median (IQR)‡	2.0 (1.5–3.0)	1.0 (1.0–2.0)	0.01	2.0 (1.0–3.0)
DAD	16 (57.1%)	0 (0.0%)	0.001	16 (41.0%)

*Corpses were admitted to the Institute of Legal Medicine, University Medical Center Hamburg-Eppendorf, Hamburg, Germany, for autopsy during 2020–22. Values are no. (%) unless otherwise noted. BMI, body mass index; DAD, diffuse alveolar damage; ICU, intensive care unit; IQR, interquartile range; NA, not applicable; PMI, postmortem interval (time elapsed from death until cooling at 4°C).
†Data for 1 patient is missing because of unknown vaccination status.
‡Microscopic changes in inflammation on the basis of a Likert-like scale: none, 0; mild, 1; moderate, 2; or severe, 3.

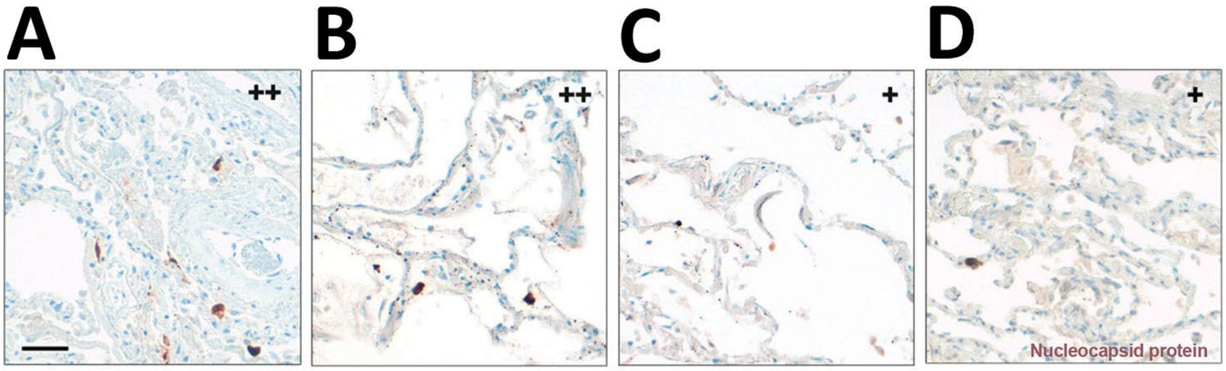

Appendix Figure. Representative immunohistochemical staining of SARS-CoV-2 nucleocapsid protein in lungs of corpses in study of new postmortem perspective on emerging SARS-CoV-2 variants of concern, Germany. Single-blind histological assessments were performed by experienced pathologists. We detected SARS-CoV-2 nucleocapsid protein in the lungs of 25/41 (61%) cases. Specimens from corpses infected with (A) non-variant of concern lineages (n=16) and variant of concern lineage (B) B.1.1.7 (Alpha, n = 6), (C) B.1.617.2 (Delta, n =4), and (D) B.1.1.529 (Omicron, n = 15) showed pulmonary virus protein abundance and pulmonary damage. Scale bar is 50 µm.
